# Test of cure and beyond: superiority of thermal ablation over LLETZ in the treatment of high-grade CIN

**DOI:** 10.1007/s00404-022-06409-3

**Published:** 2022-02-02

**Authors:** G. M. Armstrong, K. Ragupathy

**Affiliations:** 1 University of Dundee, Dundee, UK; 2 Obstetrics and Gynaecology Department, NHS Tayside, Ninewells Hospital, Dundee, UK

**Keywords:** Thermal ablation, LLETZ, CIN, Colposcopy

## Abstract

**Purpose:**

Among the treatment modalities for high-grade cervical intraepithelial neoplasia (CIN), large-loop excision of the transformation zone (LLETZ) is the commonest offered in the UK, whereas thermal ablation (TA) has not been common in several decades, despite several notable advantages. TA and LLETZ are both routinely undertaken in our colposcopy unit, and extensive follow-up data have been used to interrogate outcomes between the two modalities and determine whether one modality may be preferred over the other.

**Methods:**

Up to 8 years of follow-up data (cytology and histology) were collected for patients who have undergone LLETZ or TA and failed post-treatment test of cure (ToC). These data were analysed and used to plot Kaplan–Meier survival curves, in order to compare outcomes: negative cytology, dyskaryosis, low- and high-grade CIN and invasive squamous cell carcinoma.

**Results:**

i) Very few women treated with TA developed recurrent high-grade CIN in the follow-up period; (ii) LLETZ-treated women had a significantly higher rate of recurrence than those treated by TA; (iii) women who failed both virology and cytology components of post-treatment ToC had higher recurrence than those who failed only one, and the rate of recurrence was highest in those treated by LLETZ (> 65%).

**Conclusion:**

TA is an effective treatment of high-grade CIN, with a high chance of achieving double-negative ToC and low recurrence relative to LLETZ. We recommend the wider adoption of TA, so that young women of reproductive age have a choice of treatment with no reported adverse effects on pregnancy outcomes.

## Introduction

Cervical cancer is, both in the UK and in the wider world, a significant driver of morbidity and mortality. Among malignancies affecting women in the UK, it is the 14th commonest cancer, accounting for 2% of all new cancer cases in 2017 [1]; 3200 new cases of cervical cancer are diagnosed in the UK every year, and 850 women die of it [1]. Among gynaecological cancers specifically, it is the commonest cancer worldwide, with an incidence and mortality of over 500,000 cases and 266,000 deaths, respectively, in 2012 [2].

A relatively long stage of detectable, pre-invasive disease (cervical intraepithelial neoplasia; CIN), however, makes cervical cancer amenable to screening and early intervention; and indeed, due in large part to the successful implementation of effective screening and vaccination programmes, its incidence and mortality has been steadily declining in the developing world.

This recent success notwithstanding, questions remain about how best to treat pre-invasive disease, and what becomes of CIN treatment modalities in longer term follow-up.

### HPV testing

Persistent HPV carriage greatly predisposes women to developing cervical cancer [3, 4]. In consequence of discovering the HPV subtypes most responsible, testing women for HPV carriage has become invaluable in identifying cervical disease in its pre-invasive stage. HPV testing has been employed as a stand-alone screening tool, guiding subsequent cytology, and in conjunction with cytological screening methods [5]. It operates on the principle of detecting in cervical specimens the presence of DNA, mRNA and/or other viral markers (e.g., DNA methylation markers) associated with HPV infection. While it does not suffer the limited sensitivity of some cervical cytology methods, HPV testing can generate false and/or clinically insignificant positive results, driving unnecessary colposcopy referrals, biopsy collection and treatment of otherwise healthy women. This is both a source of unnecessary distress to patients, and a cost burden on the provision of screening, hence HPV testing is coupled with cytology both as a test of cure (ToC) following treatment or in the context of routine cervical screening.

Introduced in Scotland in 2012, ToC is undertaken 6 month post-treatment of CIN (low grade and high grade), and tests both hr HPV carriage and cytology: if both are negative, women have their next smear in 36 months. If either virology or cytology is positive, they are referred to colposcopy for further evaluation.

### Large-loop excision of the transformation zone and thermal ablation

Large-loop excision of transformation zone (LLETZ) is an excisional means of treating CIN, and while widely used in the UK, requires trained personnel and risks impaired pregnancy outcomes.

Thermal ablation (TA; previously known as “cold coagulation”), an alternative technique employing thermocoagulation, boasts several advantages over LLETZ, including reduced risk of impact on pregnancy, and ease of implementation in an outpatient context. Tissues are heated by electricity to 100–120 °C, destroying the lesion [6, 7] with few complications or adverse effects [6, 8–10]. Developed in the 1960s [11], it enjoyed a period of frequent use in the UK in the 1980s [8], but has since mostly fallen from favour in all but a few trusts, where it remains the preferred treatment method. Despite its many advantages over LLETZ, it has the notable limitation of not producing an excised specimen for histology. Its indications are non-pregnant women with any grade of CIN, as long as the entire transformation zone can be visualised, and there is no expansile crypt involvement, micro-invasive/invasive disease or glandular disease; it is, moreover, only indicated as the first treatment for CIN, and should not be used when the transformation zone has been previously treated [6–8, 12, 13].

The principal objectives of this work are to (i) evaluate and compare outcomes of failed tests of cure (HPV status and cervical cytology) following treatment of high grade CIN with TA and LLETZ; and (ii) investigate recurrent high-grade CIN and re-treatment outcomes.

## Materials and methods

### Data collection

The data used in this study was collected from patients who satisfied the following criteria: patients must have (i) been referred to and seen by our colposcopy unit within the study period (April, 2012–March 2018); (ii) had a primary histology result of high-grade CIN (CIN2 or CIN3); (iii) received treatment for high-grade CIN, either with thermal ablation or LLETZ; and (iv) failed test of cure after treatment, either due to positive hr-HPV carriage (virology), dyskaryosis (cytology) or both. Any patients who were identified to have had high-grade CIN or treatment previous to the study period were excluded.

Primary histology (CIN grade), date of histology, treatment modality (thermal ablation or LLETZ), date of treatment and test of cure results for patients were collected, then combined with follow-up data retrieved from the Scottish Cervical Call/Recall System (SCRRS) using community health index (CHI) numbers as patient-specific identifiers. These follow-up data included any abnormal cytology, recurrence of high-grade disease, (re-)treatment and treatment outcomes, as well as all instances of negative cytology.

All data were collected under Caldicott Guardian approval (SMED 19/88).

### Data analysis

These patient data were then split into two groups by treatment modality: those treated with thermal ablation and those treated with LLETZ. Within these groups, data were further split into approximate tertiles according to the duration of follow-up available to yield three subgroups each: (i) all follow-up: ≥ 627 days (thermal ablation) or ≥ 615 days (LLETZ) of follow-up; (ii) ≥ 4 years of follow-up; and (iii) ≥ 6 years of follow-up (Table 1). Outcomes of recurrent high-grade CIN (or development of invasive SCC), low-grade CIN, dyskaryosis and/or borderline cell changes, and negative cytology were noted, along with the dates of these events. In the absence of negative cytology for the full duration of follow-up, the outcome was coded as the most advanced disease developed in the follow-up period, e.g., a woman who develops CIN1 subsequent to her failed test of cure who then develops CIN3 is coded as CIN3 (with its corresponding date).

**Table 1:** Follow-up outcomes after failed tests of cure for women with high-grade CIN treated with TA and LLETZ

Follow-up groups (duration of follow-up)	TA			LLETZ		
	Group 1 (≥ 627 days; all)	Group 2 (≥ 4 years)	Group 3 (≥ 6 years)	Group 1 (≥ 615 days; all)	Group 2 (≥ 4 years)	Group 3 (≥ 6 years)
Total (failed tests of cure)	182	132	59	166	103	43
Negative cytology	119	87	40	80	47	19
No follow-up, treatment or biopsy	17	10	3	13	4	2
Positive cytology and absence of CIN (borderline cell changes, low-grade and high-grade dyskaryosis)	22	18	9	19	15	4
Borderline cell changes	17	14	7	12	9	1
Low-grade dyskaryosis	5	4	2	6	5	3
High-grade dyskaryosis	0	0	0	1	1	0
Low-Grade CIN	12	7	2	27	16	9
High-grade CIN	12	10	5	26	20	9
Invasive SCC	0	0	0	1	1	0

Follow-up time for TA was 627–2715 days; and for LLETZ, 615–2773 days

To compare the outcomes of TA and LLETZ in women who failed ToC, Kaplan–Meier survival curves were constructed. The start time (*t* = 0) was defined as the date of treatment (TA or LLETZ), failure was defined as recurrence of high-grade CIN (CIN2 or 3), and data were censored either at the end of the follow-up period or at their last date of engagement with screening/colposcopy services. Subgroups within treatment groups were further analysed: primary CIN histology (CIN2 vs CIN3) and failed test of cure outcome (virology vs cytology vs both). Data were analysed using GraphPad Prism 8 software (v8.3.0; GraphPad Software, LLC), and statistical significance was evaluated using log-rank/Mantel-Cox tests.

## Results

In the period of 01 April, 2012 to 31 March, 2018, 1008 women in Tayside had thermal ablation for high-grade CIN, of whom 909 had test of cure data available, which 182 failed (20%). In this same period, 865 women had CIN treated with LLETZ, of whom 732 had test of cure data available and 166 failed (23%). These results are summarised in Table 1.

### Cytology and histology outcomes in TA vs LLETZ

Of the women with high-grade CIN treated with thermal ablation who failed their tests of cure, only a small proportion went on to develop recurrent high-grade CIN in the follow-up period examined (Table 1). This proportion remained < 10% in even the longest follow-up group (≥ 6 years). Proportions of other outcomes, including low-grade CIN, dyskaryosis and borderline cell changes likewise remained relatively stable into the longest follow-up tertile. Negative cytology predominated among outcomes, and likewise remained between 60 and 70% across follow-up tertiles.

Likewise, in patients treated with LLETZ, outcomes also remained relatively stable across follow-up tertiles (Table 1). In this group, however, the proportion who developed recurrent disease was slightly higher than in those treated with thermal ablation, ranging from 15 to 25%. The largest proportion of outcomes in the LLETZ-treated women was still negative cytology, though in this group, it accounted for < 50%. There is one instance of invasive SCC in the LLETZ treatment group.

### Recurrence of high-grade CIN following index treatment of TA vs LLETZ

**Fig. 1:**
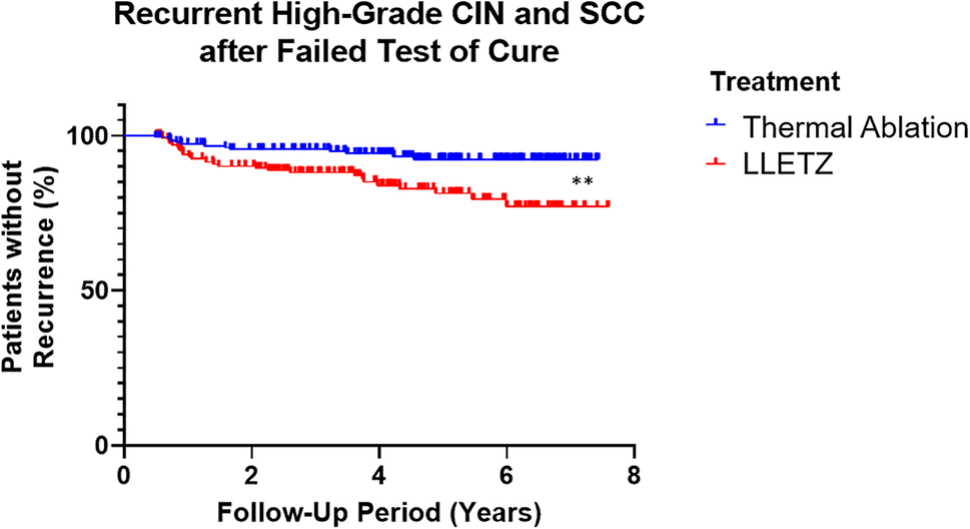
Significantly higher proportion of women who underwent LLETZ treatment and failed their test of cure went on to develop recurrent high-grade CIN. **p < 0.01; significance determined by log-rank/Mantel-Cox test

In comparing outcomes between the thermal ablation and LLETZ treatment groups, greater proportion (*p* = 0.0019) of women developed recurrent high-grade CIN in LLETZ-treated women across the follow-up period (Fig. 1).

**Fig. 2:**
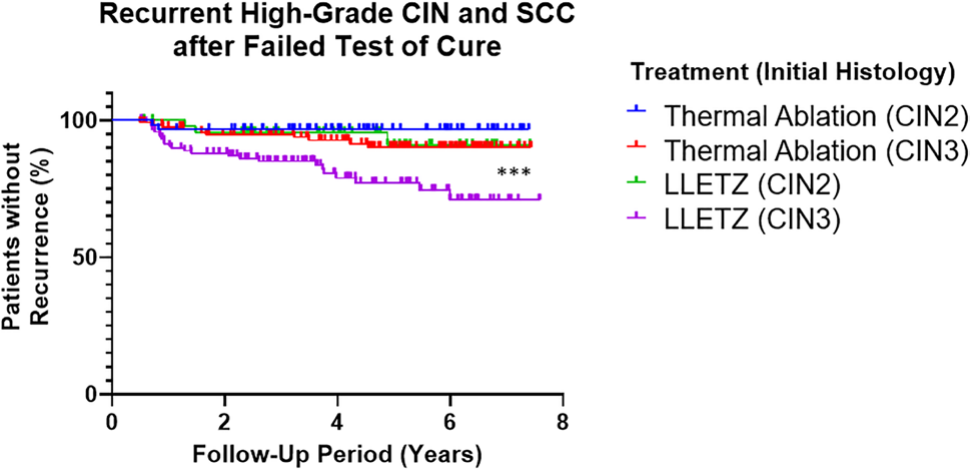
Women treated for CIN3 with LLETZ had the highest recurrence of high-grade CIN of all treatment/histology subgroups. Women treated for CIN3 had higher recurrence of disease than those treated for CIN2, in both treatment groups. ***p < 0.001; significance determined by log-rank/Mantel-Cox test

Splitting treatment groups by primary histology (CIN2 vs CIN3) yielded similar results (Fig. 2): a higher proportion (*p* = 0.0005) of women treated by LLETZ who failed their tests of cure developed recurrent high-grade CIN than those treated by TA. The highest rate of recurrent disease was seen in those treated by LLETZ for CIN3.

**Fig. 3:**
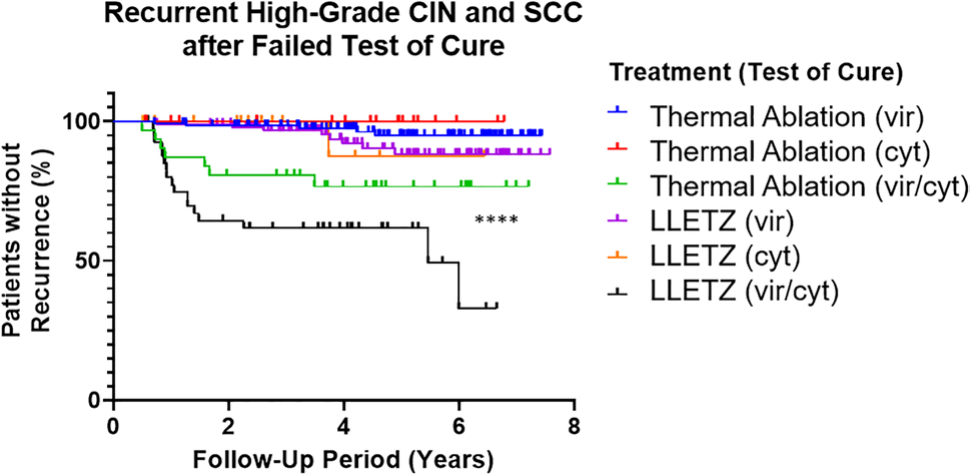
Women treated for high-grade CIN with LLETZ who failed both virology and cytology components of test of cure had the highest recurrence of high-grade CIN of all treatment/test of cure subgroups. Women who failed both test of cure components had higher recurrence of disease than those who failed only one component, in both treatment groups. ****p < 0.0001; significance determined by log-rank/Mantel-Cox test. vir indicates virology (hr-HPV carriage), cyt indicates cytology (abnormal cytology), vir/cyt indicates both (double-positive)

Investigating treatment groups split by failed test of cure subgroups (failed virology vs failed cytology vs failed virology and cytology) produced similar results (Fig. 3). Groups in which only one component of the test of cure was failed (virology *or* cytology) behaved similarly, irrespective of treatment group, but those in which both components were failed showed higher proportions of disease recurrence and showed recurrence earlier. Women treated with LLETZ with double-positive tests of cure showed the highest recurrence of disease of any group (> 65%), significantly higher than those treated with thermal ablation.

## Discussion

Historically, TA has not been as widely used as LLETZ due to concerns of inadvertently ablating micro-invasive cancer, and of inadequate depth of necrosis relative to LLETZ. Our data, however, show high rates of double-negative ToC in women treated for high-grade CIN with TA. The proportion of women who failed ToC appears to be comparable between treatment groups (20% for TA vs 23% for LLETZ) and to our knowledge, our study has the largest number of women included in the study-albeit retrospectively.

The use of TA in our centre has been first-line for 43 years, provided the following criteria are met: it must be undertaken by an appropriately trained practitioner, the transformation zone must be visualised in its entirety, and there should be no suspicion of either glandular disease or invasion [6]; any evidence of more complex histological patterns such as expansile CIN is an indication for LLETZ [14].

That outcomes of women treated with TA who failed their tests of cure should remain so stable across the follow-up period investigated (Table 1) affords reassurance to colposcopists; no evidence has been found of any significant increase in recurrent disease with increasing follow-up time. There is some suggestion that the incidence of recurrent disease increases with time in those women treated with LLETZ (Table 1), but this is difficult to appreciate in these analyses and figures.

Of greatest interest in these data is the difference in recurrent disease over time when thermal ablation and LLETZ treatments were compared in Kaplan–Meier/time-to-failure survival curve analyses: significantly more women with failed tests of cure who were treated with LLETZ went on to develop recurrent high-grade CIN in the follow-up period than those treated with thermal ablation (Fig. 1).

To demonstrate that this striking difference could not be accounted for by differences in the grade of CIN in the primary histology, treatment groups were then divided into those with a pre-treatment histology result of CIN2 and those with CIN3 (Fig. 2). Perhaps unsurprisingly, those with CIN3 showed greater recurrence of high-grade CIN in the follow-up period than those with CIN2, but the difference in disease recurrence between LLETZ-treated and TA-treated women persisted. The difference in outcomes, then, cannot be attributed to CIN grade alone. Recent studies and meta-analysis likewise identify CIN3 as being more likely to culminate in failure of ToC [15, 16], and provide further evidence in favour of thermal ablation in treatment of CIN.

Finally, to interrogate the importance of the nature of the failed tests of cure on outcomes—that is, failing the virology, the cytology or both—the treatment groups were split again according to the details of their test of cure failure (Fig. 3). Again, it is of little surprise that those women who failed the test of cure with both abnormal cytology and persistent hr-HPV carriage should fare worse than those who failed only one of the two components: this group showed much higher recurrence of disease than treatment-matched single-failure groups, and showed it much earlier in the follow-up period. Nevertheless, as before, the LLETZ treatment group showed higher recurrence than the thermal ablation treatment group, even accounting for the nature of the failed tests of cure: > 60% recurrence was observed in the LLETZ-treated double-positive group by 6 years of follow-up. A careful search of the existing literature revealed no data on the incidence of high-grade CIN specifically after double-positive ToC—we believe these to be novel—our data for single-positive ToC (either abnormal cytology or persistent hr-HPV carriage), however, are consistent with those of the literature [17, 18].

Providing there are no adverse histological parameters, we, therefore, propose that TA be considered as first-line treatment—especially in women of reproductive age, given the increased risk of pre-term labour (PTL) associated with excisional treatment identified in systematic reviews and meta-analyses [19, 20].

As compelling as the data are herein, we must acknowledge some limitations: this was a retrospective study, and in consequence of this, we were not able to collect or access data on patient risk factors (such as immunosuppression) or smoking status. The DNA rate across the study was almost 10%—comparable across LLETZ (13/166) and TA (17/182) treatment groups. While some effort was made to investigate the effect of primary histology on recurrent high-grade CIN in the two treatment groups (Fig. 2), these analyses were not able to consider any additional histological features, such as expansive crypt involvement. Since this feature may preclude the use of TA as a treatment, it must also be acknowledged that this presents the possibility of selection bias which was not adequately controlled for by separating primary histology (CIN2 vs CIN3) in treatment groups.

We acknowledge that the above adverse clinical and pathological parameters could contribute to the persistence of high-grade CIN, and hence suggest that TA is at least as effective as LLETZ in the treatment of high-grade CIN; we intend to explore, however, the outcomes of LLETZ vs TA in future with standardised histological parameters.

The follow-up data and the Kaplan–Meier survival curves generated (Figs. 1, 2, 3) have only examined the recurrence of *high-grade* CIN and invasive SCC; recurrence of low-grade CIN or the persistence of abnormal cytology were not included in the analyses, but are noted in the follow-up outcomes in previous graphs (Table 1).

## Conclusions

Thermal ablation is an effective treatment of high-grade cervical intraepithelial neoplasia, with a high chance of achieving double-negative test of cure outcomes. Providing strict clinical criteria are adhered to when selecting TA as the mode of primary treatment, the recurrence of HG CIN is low. TA has to be adopted more widely, so that young women of reproductive age have a choice of treatment with no reported adverse effects on pregnancy outcomes.
